# Short- and Long-Term Effects of Drought on Selected Causes of Mortality in Northern Bangladesh

**DOI:** 10.3390/ijerph19063425

**Published:** 2022-03-14

**Authors:** Intekhab Alam, Shinji Otani, Abir Nagata, Mohammad Shahriar Khan, Toshio Masumoto, Hiroki Amano, Youichi Kurozawa

**Affiliations:** 1 Division of Health Administration and Promotion, Department of Social Medicine, Faculty of Medicine, School of Medicine, Tottori University, Yonago 683-8503, Japan; ian.alam03@gmail.com (I.A.); tmasumoto@tottori-u.ac.jp (T.M.); h-amano@tottori-u.ac.jp (H.A.); kurozawa@tottori-u.ac.jp (Y.K.); 2 International Platform for Dryland Research and Education, Tottori University, Yonago 680-0001, Japan; khanshahriar@tottori-u.ac.jp; 3 Department of Regenerative Dermatology, Graduate School of Medicine, Osaka University, Osaka 565-0871, Japan; abir.med@osaka-u.ac.jp

**Keywords:** drought, public health, mortality, SPI, SPEI, SVRS

## Abstract

Drought has exacerbated morbidity and mortality worldwide. Here, a time series study was conducted in northern Bangladesh to evaluate the impact of drought on selected causes of mortality during 2007–2017. Rainfall and temperature data from six meteorological stations were used to analyze drought and non-drought periods and to categorize mild, moderate, severe, and extreme drought based on the 3-month and 12-month Standardized Precipitation Index (SPI) and Standardized Precipitation Evaporation Index (SPEI). A generalized linear model with Poisson regression with log link, a negative binomial with log link, and a zero-inflated Poisson model were used to determine associations between drought severity and mortality. The SPI and SPEI produced slightly different analysis results. Compared with the SPEI, the SPI showed a stronger and more sensitive correlation with mortality. The relative risk for respiratory disease mortality was high, and Saidpur was the most vulnerable area. Health care expenditure was negatively associated with mortality. High temperatures during the drought period were associated with suicide-related mortality in Rajshahi. The impact of drought on mortality differed with small changes in climate. The findings of this study improve our understanding of the differences between the two most used drought indicators and the impact of drought on mortality.

## 1. Introduction

Climate change poses an ongoing challenge for countries [1]. Higher temperatures cause increased variability in precipitation and extreme weather events, such as droughts, floods, and seasonal shifts, which are associated with increased greenhouse gas emissions [2,3]. Weather, climate, and water crises accounted for 50% of all disasters, 45% of recorded deaths, and 74% of reported economic losses from 1970 to 2019. Developing countries accounted for more than 91% of these deaths. Drought (650,000 deaths), storms (577,232 deaths), floods (58,700 deaths), and extreme temperatures (55,736 deaths) were the disasters that caused the greatest loss of life during this period [4].

Drought is the most complex natural phenomenon among all natural disasters and has far-reaching impacts [5]. It is caused by a lack of precipitation [6] and is divided into four types. Meteorological drought refers to rainfall deficit, hydrological drought refers to low water levels in streams, agricultural drought refers to low moisture levels in the soil, and socioeconomic drought is when water demand exceeds the supply [7]. Meteorological drought can be assessed by the annual rainfall [8]. Yearly precipitation that is 25–50% lower than average is considered moderate drought, while yearly precipitation that is 50% lower than average is considered severe drought. A year is termed a drought year if the country as a whole receives less than 20% of its average annual precipitation [9].

Droughts have become more common and severe in many parts of the world because of global warming and climate change owing to higher temperatures and changes in rainfall patterns [10,11]. However, the biological mechanisms by which drought impacts human well-being are unclear. Drought might impact disease prevalence through facilitating exposure by increasing airborne dust or smoke from forest fires and altering the growth and spread of pollen and allergenic fungal spores [12]. One of the few studies on the health effects of drought, which was conducted in the USA, found that under more severe drought conditions, adults over 65 are at higher risk of death; in particular, those living in areas that have experienced less extreme drought conditions are at greater risk of cardiovascular disease and mortality [13]. Several studies in Spain have suggested that short-term drought with high temperatures and air pollution impact selected causes of mortality [14,15,16]. Our previous study conducted in the northernmost region of Bangladesh also revealed the impact of short-term extreme drought on natural cause and respiratory disease-related mortality [17].

As the Intergovernmental Panel on Climate Change points out, some low- and middle-income countries are exposed to the effects of climate change. Bangladesh is a middle-income country, and various studies have found that the rate of warming in Bangladesh is faster than the global average and will continue in the next decades [2,18,19]. Drought is a common occurrence in several parts of Bangladesh; nevertheless, owing to its high precipitation variability, the northwest region is the most drought-prone [20]. This region receives less rainfall than the rest of the country [21] and has low moisture-retention capacity and infiltration rate characteristics [22], which keep the area relatively dry and increase the chance of drought. Severe droughts have occurred in Bangladesh (in 1951, 1961, 1975, 1979, 1981, 1982, 1984, 1989, 1994, 1995, and 2000), affecting approximately 53% of the population [23]. Drought has led to the contamination of existing water supplies, especially rivers and stagnant ponds, and dysentery and diarrhea in Bangladesh have increased considerably because of the use of non-potable drinking water [24].

Health expenditure by the government, non-governmental organizations, health insurance companies, and private financing (i.e., out of pocket) all play a role in a country’s health status and mortality rate [25]. In Bangladesh, 3% of gross domestic product (GDP) is spent on health care, with the public sector accounting for less than one-third. The country’s total health expenditure (THE) is USD37 per capita per year [26]. According to a comparison of THE as a percentage of GDP across South Asian Association for Regional Cooperation nations in 2014, Bangladesh was the second lowest in the region. Nevertheless, in the past two decades, the overall health of Bangladesh’s population has significantly improved. Between 2000 and 2017, men’s life expectancy increased by 7 years, while women’s life expectancy increased by 10 years (SVRS 2017). One study found that health care expenditure potentially reduces maternal and infant mortality in lower- and middle-income countries [27].

In our previous study, we investigated the effects of drought on mortality in northern Bangladesh. It was a regional study, covering the Nilphamari, Dinajpur, and Rangpur areas in the north of the country, and did not consider different drought indices and included only three causes of death [17]. However, because precipitation varies greatly in this region, and because there are climatic and meteorological differences among geographical locations in northern Bangladesh, the conclusions reached for the northernmost part of the country cannot be conclusively applied to the rest of the region. Thus, in the current study, we investigated the whole of northern Bangladesh to gain a better understanding of the short- and long-term consequences of drought on specific causes of mortality over the 2007–2017 period. We also compared the performance results of two different drought indices to determine which proxies are best for estimating health risks.

## 2. Materials and Methods

In this retrospective ecological study, we assessed the short- and long-term effects of drought on natural cause-, circulatory-, respiratory-, infectious disease-, and suicide-related mortality in northern Bangladesh from 2007 to 2017. The primary objective was to analyze the impact of drought as measured by the 3- and 12-month Standardized Precipitation Evapotranspiration Index (SPEI-3, SPEI-12) and Standardized Precipitation Index (SPI-3, SPI-12) on each type of mortality. The secondary objective was to identify which drought index is more suitable for our study area.

### 2.1. Study Area

**Figure 1:**
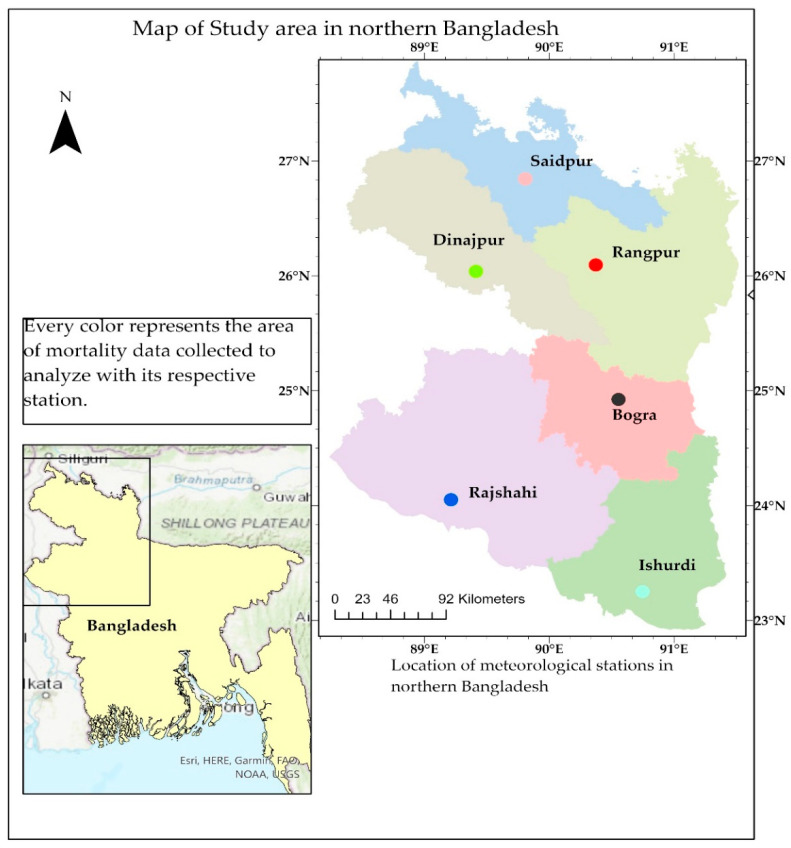
Study area and meteorological station locations in northern Bangladesh.

The study area was the northern region of Bangladesh. The Rajshahi and Rangpur divisions, consisting of 16 districts, make up northern Bangladesh. To compare mortality statistics with drought periods, the study area was divided into six parts, based on nearest place to a weather station, each represented by one meteorological station and for which mortality data were collected (Figure 1). The six Bangladesh Meteorological Department (BMD) weather stations in Rangpur and Rajshahi division provided the climate data (rainfall, temperature, and humidity) for this study. Table 1 shows the study area’s geographical situation [26,28,29,30,31].

### 2.2. Meteorological Data

Data on monthly precipitation, temperature, and relative humidity were collected for the 1989–2018 time period from the six BMD meteorological stations in the study area and used to acquire insight into drought characteristics. However, the analysis in the current investigation used time series data from the 2007–2017 time period.

### 2.3. Mortality Data

Mortality data covering 2007–2017 were obtained through the Sample Vital Registration System (SVRS). SVRS, a core operation of the Bangladesh Bureau of Statistics (BBS), collects critical information, such as births and deaths, from a sample population of approximately 1 million people. There are 1000 primary sample units, each containing 250 households with an average of 4.5 individuals [28]. THE data were collected from a Bangladesh national health account publication [26].

We also collected 2007–2017 daily mortality data from across the study area. SVRS listed 64 different causes of death for this dataset, which we classified by station and cause. To compare with the monthly drought period, we categorized daily mortality and converted it to monthly mortality. We chose five causes of death for the study population and classified them using the International Classification of Diseases 10 (ICD-10): natural cause mortality (A00–R99), circulatory-related mortality (I00–I99), respiratory-related mortality (J00–J99), infectious disease-related mortality (A00–B99), and intentional self-harm (suicide) (X60–X84).

### 2.4. Analyzing Drought

Drought indicators are important instruments for tracking and defining drought [32] and assessing its many effects [33,34]. There are various methods for assessing and monitoring drought conditions [35], including the Palmer drought severity index [36], SPI [8], and SPEI [37]. The SPI and SPEI have several timeframes, allowing them to represent different types of droughts and to better reflect fluctuations in drought features [38]. The SPEI is calculated based on the accumulated difference between precipitation (P) and potential evapotranspiration (PET), which can comprehensively reflect changes in the surface water balance [39]. The SPI considers only precipitation, is easy to calculate, and has temporal and spatial robustness [40,41]. For a realistic assessment of drought in the context of global warming, the increase in evaporation produced by warming is not negligible. As a result, the SPEI outperforms the SPI in drought monitoring [42]; however, its applicability in arid regions may be limited [43]. The differences in drought monitoring between the SPI and SPEI, as well as their robustness in the context of global climate change, are still open to debate. In the current study, the SPI and SPEI were calculated at different timescales using the SPEI package in R [44]. The potential evapotranspiration variable was initially evaluated using the Hargreaves method [45] to produce the SPEI series. Fitting the P and P-PET series to a suitable probability distribution is required to calculate these indicators. The SPI [8] and SPEI [37] are defined by converting the fitted series into standardized values.

Drought was calculated using monthly data from 1989 to 2018. To assess short- and long-term drought, both indices were calculated for 3 and 12 months cumulatively. Studies have suggested that the application of the SPI and SPEI series at a 3-month scale (short-term) produces a high temporal frequency of periods of drought and moisture and that the 12-month SPI is better at indicating long term drought results, better also than for 36 and 48 months. Therefore, in this study, we used 3-month SPI/SPEI to assess short-term drought and 12-month SPI/SPEI to assess long-term drought [8,46]. Table 2 contains the actual SPI categories used for drought calculation based on severity. Agnew et al., however, proposed thresholds to ensure that the SPI categories were more appropriate, the thresholds also being suitable for SPEI categories, and this has been applied by many researchers subsequently. Based on this, the onset of a drought episode was defined as when the index value falls below zero and the episode ends when the index returns a positive value; furthermore, its severity could be quantified by applying certain thresholds (based on a normal distribution). Drought conditions were rated from 0 to 4, where 0 is ‘no drought’ and 4 is ‘extreme drought’ [13,14,47]. The revised values used in this study for the SPI and SPEI drought categories based on severity are described in Table 2. To be able to ascertain the influence of each drought severity level in our study, we used this classification of drought and did not use a direct link between negative and positive SPI values. We included −0.85 < SPI/SPEI ≤ 0 as mild drought because, while mild drought may not have a significant impact on agriculture and has not typically been included in previous health-related research, mild drought might have a considerable influence on health impact analyses.

### 2.5. Statistical Analysis

Two distinct analyses were performed to estimate the relative risk (RR) and impact on mortality associated with exposure to drought. This included monthly deaths from all causes and cause-specific mortality (e.g., cardiovascular, respiratory, infectious disease, suicide), and the duration of the drought with respect to its severity (i.e., mild, moderate, severe, and extreme, with no drought as a reference). We first analyzed drought using R software. We then defined drought as a categorical variable, characterizing the drought severity as 0, 1, 2, 3, 4, based on the value of SPI and SPEI. Here, 0 represents no drought, 1 represents mild drought, 2 is moderate drought, 3 is severe drought and 4 is extreme drought. This categorical variable was used as the independent variable against dependent variable mortality data. We used a generalized linear model based on a Poisson distribution to define the relationship between mortality and drought severity based on the SPI and SPEI categories. The consequences of drought and non-drought periods were clarified by these analyses. Two models were created in which model 1 consisted of drought, mortality, temperature, and humidity. As drought is closely related to temperature and humidity, it was appropriate to use temperature and humidity as covariates. This enabled assessment of the direct impact of drought on mortality. In model 2, along with model 1, we used seasonality impact and health expenditure as the covariates, providing the result of adjusted drought impact on mortality. The two different models applied in this study were constructed to determine the adjusted and unadjusted impacts of drought. Only the RR results are included in this paper for Model 2. To assess the effects of seasonality, we also conducted quarterly assessments using the following quarters: Q1 (December–February), Q2 (March–May), Q3 (June–August), and Q4 (September–November). Statistical significance was defined as a p value of less than 0.05. For each cause of death and each timescale for both drought indices, we used Poisson regression with log link, negative binomial with log link, and zero-inflated Poisson regression (ZIP) to analyze the impact on mortality, with the most appropriate results being used for this study. Goodness of fit for high and low dispersion, an omnibus test, Akaike’s information criterion (AIC), and Bayesian information criterion (BIC) values were checked for every analysis to determine over- and under-exposure of each test. The IBM SPSS Statistics 25 package (IBM Corp., Armonk, NY, USA) was used to conduct all the analyses. The R software package version 3.3.6 (https://www.r-project.org/, accessed on 11 October 2020) was used to conduct the drought analysis.

### 2.6. Ethical Approval

This study did not require research ethics approval because publicly available, anonymized aggregate data were used for all analyses.

## 3. Results

### 3.1. Drought Statistics for Northern Bangladesh

The total number of drought months and percentages during the study period (2007–2017) based on drought severity (no drought, mild drought, moderate drought, severe drought, and extreme drought) are shown in Table 3. There were very few differences in the drought periods as defined by the SPI and SPEI. The highest percentage of no drought based on the SPI-3 and SPEI-3 was in Rajshahi (46.97% and 45.45%, respectively). The highest percentages of no drought based on the SPI-12 and SPEI-12 were in Bogra (35.61% and 32.57%, respectively). Compared with the rest of the areas, the percentage of mild drought was much higher based on all indices in Dinajpur, Rangpur, and Saidpur. The SPI-12 and SPEI-12 resulted in more drought months than the SPI-3 and SPEI-3. The moderate and severe drought results were quite similar for both indices. However, there was a clear difference in the results for extreme drought. The SPI showed much longer durations of extreme drought in Rajshahi, Bogra, and Ishurdi compared with the SPEI. Additionally, the SPI-12 showed a higher percentage of extreme drought than the SPI-3. There was a higher number of mild and moderate drought months in Rangpur, Saidpur, and Dinajpur (northwestern part of the study area). The number of severe and extreme drought months was higher in Rajshahi, Bogra, and Ishurdi. There were no months of extreme drought in Dinajpur according to both indices and timescales. According to the SPI-12 and SPEI-12, there was no extreme drought in Rangpur or Saidpur. The largest difference between the SPI and SPEI calculations was found for the Rajshahi and Ishurdi stations during extreme drought. For Rajshahi, the SPI-12 showed 6.82% extreme drought during the study period, but the SPEI-12 showed only 0.76%. Similarly, for Ishurdi, the SPI-12 showed 6.06% extreme drought, but the SPEI-12 showed 0%.

### 3.2. Drought Effects on Selected Causes of Mortality in Northern Bangladesh (Model 1)

Table 4 shows the individual cause and total mortality in northern Bangladesh during 2007–2017 for selected causes of mortality. The impact of drought on mortality was mainly found in the short-term drought period. The impact varied by climate and region. The two drought indicators gave slightly different results, with the SPI indicating a higher level of impact on selected causes of mortality.

On the basis of the SPI-3, extreme drought was associated with respiratory and infectious disease mortality in Bogra, with natural cause and respiratory disease mortality in Ishurdi, and with natural cause and infectious disease mortality in Saidpur. On the basis of the SPI-3, mild drought was associated with cardiovascular disease mortality in Dinajpur. According to the SPI-3, drought was negatively associated with mortality in Rajshahi and Rangpur. According to the SPI-12, drought was negatively associated with mortality in all six areas. The SPI-12 showed that moderate drought was significantly associated with suicide-related mortality only in Saidpur (Table 5, Table 6, Table 7, Table 8, Table 9 and Table 10).

The SPEI-3 showed that severe drought (ZIP model) was associated with suicide mortality in Bogra. In Dinajpur, the SPEI-3 showed that mild drought was associated with cardiovascular disease and that severe drought was associated with respiratory disease. In Ishurdi, the SPEI-3 and SPI-3 both showed that drought was associated with natural cause and respiratory disease mortality. In Rangpur, the SPEI-3 showed that severe drought was associated with natural cause mortality. Rajshahi and Saidpur did not show any impacts based on the SPEI-3. The SPEI-12 revealed a negative impact for all areas. The results based on the SPI and SPEI were different for Bogra, Dinajpur, and Saidpur. The RR of respiratory disease in Bogra and Ishurdi, and the RR of suicide in Saidpur, were significantly higher than in the other areas (Table 5, Table 6, Table 7, Table 8, Table 9 and Table 10).

During drought periods, the temperature and humidity also showed significant impacts on mortality. Humidity was associated with infectious disease mortality according to the SPI-3/12 and SPEI-3/12 in Bogra and Dinajpur, and with suicide in Saidpur according to the SPI-3. Temperature was associated with suicide mortality in Rajshahi according to all indices (Supplementary Tables S1–S6).

### 3.3. Drought Associated with Selected Causes of Mortality in Northern Bangladesh (Model 2)

**Figure 2:**
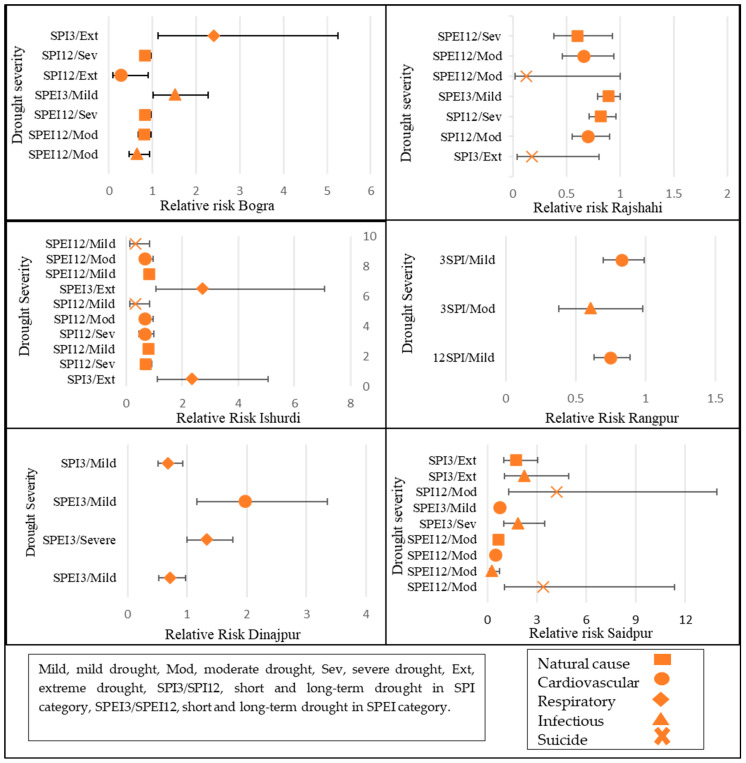
Relative risk for significant associations between drought and mortality from 2007 to 2017.

Model 2 showed slightly different results from Model 1 based on the SPI-3. In Bogra, on the basis of the SPI-3, extreme drought was associated with respiratory disease mortality (RR = 2.42, 95% CI, 1.12; 5.25), and on the basis of the SPEI-3, mild drought was associated with infectious disease mortality (RR = 1.53, 95% CI, 1.02; 2.28) (Figure 2). In Dinajpur, according to the SPEI-3, mild drought was associated with cardiovascular disease (RR = 1.33, 95% CI, 1.00; 1.77) and severe drought was associated with respiratory disease (RR = 1.97, 95% CI, 1.16; 3.35). In Ishurdi, for both the SPI-3 and SPEI-3, extreme drought was associated with respiratory disease mortality (RR = 2.36, 95% CI, 1.10; 5.05, RR = 2.73, 95% CI, 1.05; 7.08, respectively). In Rajshahi and Rangpur, there were no positive associations based on any of the indices. In Saidpur, on the basis of the SPI-3, extreme drought was associated with natural cause and infectious disease mortality (RR = 1.75, 95% CI, 1.00, 3.05; RR = 2.25, 95% CI, 1.03; 4.93, respectively). For the SPI-12, moderate drought was associated with suicide-related mortality, with a very high relative risk (RR = 4.23, 95% CI, 1.28; 13.93). On the basis of the SPEI-3, severe drought was associated with infectious disease mortality (RR = 1.86, 95% CI, 1.00; 3.46). According to the SPEI-12, moderate drought was also associated with suicide-related mortality (RR = 3.40, 95% CI, 1.02; 11.36). Short-term extreme drought had a higher RR then long-term drought. Saidpur was the most vulnerable area in terms of drought impact.

In Bogra, on the basis of the SPI-3 and SPEI-3, humidity was associated with natural cause mortality. Temperature had a negative impact on mortality in all areas. THE was negatively associated with mortality in all areas (Supplementary Tables S7–S11).

## 4. Discussion

In this detailed study in northern Bangladesh, we found differences in climate among the included areas, particularly between the two divisions of Rajshahi and Rangpur. The northernmost part of the country (Rangpur, Saidpur, and Dinajpur) had more rainfall and lower temperatures than the rest of the northern part (Rajshahi, Bogra, and Ishurdi). These climatic differences led to different drought characteristics, as well as different impacts on mortality. The areas with less rainfall had more severe and extreme drought than areas receiving more rainfall. Additionally, we found that short-term drought had a stronger impact on mortality than long-term drought. Corroborating these findings, several studies in Spain have found that short-term drought is similarly associated with selected causes of daily mortality [14,15,16].

It was difficult to distinguish between the suitability of the SPI and SPEI for analyzing our study area, but we found that the SPI had stronger associations with mortality than the SPEI in both Models 1 and 2. Other studies have also found slight differences between the SPI and SPEI; however, they found that the SPEI has stronger associations with mortality than the SPI [48]. We also found that, during short-term drought, Bogra, Ishurdi, and Saidpur were vulnerable to extreme drought only. Natural cause, respiratory disease, and infectious disease mortalities were only associated with extreme drought in the short-term drought period. Some studies have suggested that severe droughts inflict more damage, which explains the impact of extreme drought on natural cause mortality and the low impact on mortality from mild and moderate drought [13,14,17,49]. According to a US study, among people aged 65 and older, severe drought increased the risk of mortality, but decreased the risk of respiratory admissions throughout all analyzed drought periods [13]. However, our findings were not similar; on the basis of the SPI-3, Dinajpur was vulnerable to mild drought associated with cardiovascular disease mortality, and severe drought was associated with respiratory disease mortality. Infectious disease mortality and suicide-related mortality were associated with both the drought period and temperature or humidity. Bogra and Saidpur were the most vulnerable to infectious disease- and suicide-related mortality during drought periods. Regarding long-term drought, the only identified association was suicide during moderate drought in Saidpur.

We found that the RR of respiratory disease mortality was much higher in Bogra than in the other areas. Bogra, Dinajpur, and Ishurdi were all impacted by respiratory disease mortality. Several studies have collectively examined other environmental factors (e.g., heat waves, forest fires, and pollution) that have significant associations with mortality, particularly among vulnerable populations. Extreme drought is linked to high temperatures [50,51], which results in dry soil, deforestation, the prevalence and severity of forest fires and dust storms, and other situations that degrade the air quality [52]. All of these drought-related phenomena increase dust, which transports pathogens and particulate matter, as well causing an increase in the release of hazardous aerosols into the atmosphere, all of which can have negative health consequences. The high RR associated with respiratory disease mortality found in our study can be explained by the longer dry season in Dinajpur and the severe drought in Bogra and Ishurdi, which resulted in poor air quality.

Regarding covariates, we found that temperature had a negative impact on mortality in all areas. Humidity was significantly associated with infectious disease mortality in Bogra and Dinajpur at both timescales. Similarly, previous research in Bangladesh has linked low temperatures to a significant increase in natural cause mortality and to deaths due to cardiovascular, respiratory, and neonatal causes [53,54,55]. Seasonal low absolute temperatures are not the cause of winter excess mortality, rather, a seasonal drop in average temperature is the cause of winter excess mortality [54,56]. Another study suggested that warm climate countries have higher excess winter deaths owing to inadequate housing and ineffective clothing protection against cold [57].

We found that in Rajshahi, during drought periods, higher temperatures were associated with suicide-related mortality. This important finding differs from those of previous studies. In our dataset, the highest temperature was recorded in 2016 in Rajshahi [31], which could be why we found the connection with suicide. Another finding was that THE was negatively associated with mortality, meaning that, as health care expenditure increased, mortality decreased. In support of this finding, a previous study found that health care expenditure potentially reduces maternal and infant mortality in lower- and middle-income countries [27].

We found that drought had the least impact on mortality in Rajshahi and Rangpur. According to a report from the Economic Census in Bangladesh, focused public investment and special credit programs aided the expansion of non-farm activity in these areas, which has helped to alleviate the drought crisis in recent years [58]. In addition, medical care and facilities are more accessible in Rangpur and Rajshahi. These two factors—an improved economy and better medical facilities—likely play a role in reducing the impact of drought on mortality. Furthermore, the highest level of per capita government spending is in the Rajshahi division [26], which might be another reason why no association was found between drought and mortality. Future studies should consider hospital admissions and morbidity in Rajshahi to gain a greater understanding of drought impacts. Our previous study in northern Bangladesh found an impact from extreme drought in Rangpur, which is a slightly different result than found in the current study [17]. The current study differs mainly because of the larger study area and greater amount of mortality data.

While interpreting the results, the following limitations should be taken into consideration. First, numerous factors, such as a population’s vulnerability, health status, gender, age, socioeconomic features, and access to and use of water, can amplify the effects of drought and reinforce the dangers associated with its frequency [59,60,61]. However, we were unable to account for all these factors in our investigation. Other limitations of this study are that the mortality data were collected at random, and the mortality classifications were broad and did not adhere to the International Classification of Diseases guidelines. In addition, there were very few weather stations in our study area, and there may have been some variation in drought conditions in areas furthest from the stations. Another limitation was that we did not consider flood data in this study, as well as river and drainage area data this could play a vital role regarding drought. These constraints should be considered in future research. Finally, we have been unable to examine some variables that could affect vulnerability to drought, such as individual socioeconomic status, previous health conditions and adaptive capacity of the population, sex, and age. Future research with a large cohort study in northern Bangladesh considering all these features alongside droughts and mortality can widen our understanding of the impact of drought.

On the basis of our study results, we found different health risks associated with drought that need to be addressed by the government and local authorities to mitigate the situation. We believe this study will help authorities to deploy specific measures to each area to combat the health risks more efficiently. Because there is a good understanding of the regional characteristics, authorities may be able to allocate medical resources more efficiently, which could save both lives and money. Additionally, the local health care workforce should be aware of the health risks of drought for individual areas, and the government should increase its support for health care expenditure in the most vulnerable area, Saidpur. To reduce health risks associated with drought’s numerous health impacts, it is vital to support comprehensive and visionary policies based on drought prevention, mitigation, and adaptation. It is critical to create comprehensive plans that include actions to mitigate the effects of drought. Surveillance systems and the action plans that go with them should be created with the conditions of a particular region in mind, particularly the local climate and the health needs of the population.

## 5. Conclusions

In this study, the SPI and SPEI were found to have minimal differences when analyzing drought; however, in our study area, the SPI was more strongly associated with mortality. Short-term drought, especially extreme drought based on the SPI-3, had the strongest association with mortality. Temperature and humidity also play an important role in exacerbating mortality during drought periods. Drought varies regionally depending on small changes in climate. Thus, it is important to understand the drought characteristics and impacts in a given location and consider tailored approaches for dealing with them. The findings of this study will be useful in examining the impact of drought over various timescales and for different causes of mortality in other drought-prone areas and countries affected by climate change.

## Figures and Tables

**Table 1:** Characteristics of the study area.

Station	Area Covered by Meteorological Station (km^2^)	Study Population Selected from SVRS	Yearly Average Rainfall, 2007–2017 (mm)	Monthly Average Temperature, 2007–2017 (°C)	Monthly Average Humidity, 2007–2017 (%)	Per Capita Health Care Expenditure (US $) (2017)
Bogra	3884.40	37,125	1554.72	30.94	76.94	37.07
Ishurdi	4868.92	38,250	1370.76	31.38	78.35	37.07
Rajshahi	9441.29	56,250	1258.5	31.6	78.65	37.07
Dinajpur	5226.04	33,750	1641.12	30.24	78.07	29.49
Rangpur	6824.87	75,375	1961.52	29.82	79.32	29.49
Saidpur	4295.70	41,625	1825.5	30.37	78.51	29.49

SVRS, Sample Vital Registration System.

**Table 2:** Drought characteristics based on actual and revised values.

Drought Category	Actual SPI Value	Revised SPI Value Used in This Study	Revised SPEI Value Used in This Study
No drought	>0	>0	>0
Mild drought	−0.99 ≤ 0	−0.85 ≤ 0	−0.85 ≤ 0
Moderate drought	−1.49 ≤ −1	−1.27 ≤ −0.85	−1.27 ≤ −0.85
Severe drought	−1.99 ≤ −1.50	−2.05 ≤ −1.28	−2.05 ≤ −1.28
Extreme drought	≤−2.00	≤−2.06	≤−2.06

**Table 3:** Number of drought months and percentage during the study period according to drought severity.

Station Name	Drought Category	SPI-3 No. Months (%)	SPI-12 No. Months (%)	SPEI-3 No. Months (%)	SPEI-12 No. Months (%)
BOGRA	ND	52 (39.3)	47 (35.61)	51 (38.60)	43 (32.57)
	MD	41 (31.06)	32 (24.24)	47 (35.60)	32 (24.24)
	MOD	22 (16.67)	19 (14.39)	19 (14.39)	25 (18.93)
	SD	16 (12.12)	29 (21.97)	15 (11.36)	31 (23.48)
	ED	1 (0.76)	5 (3.79)	0 (0.00)	1 (0.76)
DINAJPUR	ND	55 (41.67)	22 (16.67)	52 (39.3)	20 (15.15)
	MD	53 (40.15)	84 (63.64)	53 (40.15)	82 (62.12)
	MOD	16 (12.12)	16 (12.12)	21 (15.90)	19 (14.39)
	SD	8 (6.06)	10 (7.58)	6 (4.54)	11 (8.33)
	ED	0 (0.00)	0 (0.00)	0 (0.00)	0 (0.00)
ISHURDI	ND	58 (43.94)	43 (32.58)	57 (43.18)	36 (27.27)
	MD	39 (29.55)	49 (37.12)	37 (28.03)	53 (40.15)
	MOD	17 (12.88)	18 (13.64)	20 (15.15)	21 (15.90)
	SD	16 (12.12)	14 (10.61)	17 (12.87)	22 (16.67)
	ED	2 (1.52)	8 (6.06)	1 (0.76)	0 (0.00)
RAJSHAHI	ND	62 (46.97)	43 (32.58)	60 (45.45)	35 (26.52)
	MD	35 (26.52)	56 (42.42)	37 (28.03)	52 (39.3)
	MOD	22 (16.67)	10 (7.58)	21 (15.90)	22 (16.67)
	SD	11 (8.33)	14 (10.61)	13 (9.84)	22 (16.67)
	ED	2 (1.52)	9 (6.82)	1 (0.76)	1 (0.76)
RANGPUR	ND	55 (41.67)	36 (27.27)	54 (40.90)	30 (22.72)
	MD	53 (40.15)	72 (54.55)	51 (38.60)	77 (58.33)
	MOD	15 (11.36)	14 (10.61)	16 (12.12)	13 (9.84)
	SD	7 (5.30)	10 (7.58)	10 (7.57)	12 (9.09)
	ED	2 (1.52)	0 (0.00)	1 (0.76)	0 (0.00)
SAIDPUR	ND	56 (42.4)	23 (17.42)	52 (39.3)	26 (19.69)
	MD	55 (41.67)	90 (68.18)	53 (40.15)	78 (59.09)
	MOD	16 (12.12)	9 (6.82)	18 (13.63)	17 (12.87)
	SD	3 (2.27)	10 (7.58)	9 (6.81)	11 (8.33)
	ED	2 (1.52)	0 (0.00)	0 (0.0)	0 (0.00)

ND, no drought; MD, mild drought; MOD, moderate drought; SD, severe drought; ED, extreme drought; SPEI-3/12, 3- and 12-month Standardized Precipitation Evapotranspiration Index; SPI-3/12, 3- and 12-month Standardized Precipitation Index.

**Table 4:** Individual and total number of deaths during 2007–2017 for select causes of mortality.

Station Name	Natural Cause Mortality	Cardiovascular Disease Mortality	Respiratory Disease Mortality	Infectious Disease Mortality	Suicide
Bogra	1415	285	279	131	19
Ishurdi	1548	417	229	131	33
Rajshahi	3130	933	457	287	47
Dinapur	1340	323	303	136	22
Rangpur	3003	816	551	292	45
Saidpur	1585	366	370	154	24
Total	12,021	3140	2189	1131	190

**Table 5:** Statistically significant associations between drought and mortality in Bogra, 2007–2017.

Drought Index	Mortality Category	Drought Severity	B	Lower	Upper	P	EXP(B)	Lower	Upper
SPI-3	RD	Extreme	1.17	0.42	1.92	0.00	3.22	1.52	6.81
	ID	Moderate	0.47	0.00	0.94	0.05	1.60	1.00	2.56
SPI-12	NC	Severe	−0.22	−0.37	−0.07	0.01	0.80	0.69	0.94
	CD	Extreme	−1.23	−2.38	−0.08	0.04	0.29	0.09	0.93
	RD	Severe	−0.33	−0.64	−0.01	0.04	0.72	0.53	0.99
	RD	Moderate	−0.41	−0.79	−0.03	0.03	0.66	0.45	0.97
	RD	Mild	−0.42	−0.74	−0.09	0.01	0.66	0.48	0.91
SPEI-3	SU	Severe	2.45			0.01	(ZIP model)		
SPEI-12	NC	Severe	−0.24	−0.40	−0.09	0.00	0.78	0.67	0.91
	NC	Moderate	−0.24	−0.41	−0.08	0.00	0.79	0.67	0.93
	RD	Severe	−0.35	−0.66	−0.04	0.03	0.70	0.52	0.96
	RD	Moderate	−0.49	−0.84	−0.14	0.01	0.61	0.43	0.87
	RD	Mild	−0.51	−0.84	−0.18	0.00	0.60	0.43	0.84

No drought used as reference. NC, natural cause; CD, cardiovascular disease; RD, respiratory disease; ID, infectious disease; SU, suicide; B, unstandardized beta; P, statistical significance.

**Table 6:** Statistically significant associations between drought and mortality in Dinajpur, 2007–2017.

Drought Index	Mortality Category	Drought Severity	B	Lower	Upper	P	EXP(B)	Lower	Upper
SPI-3	CD	Mild	0.30	0.02	0.58	0.035	1.348	1.021	1.780
SPEI-3	CD	Mild	0.30	0.02	0.58	0.04	1.35	1.02	1.79
	RD	Severe	0.47	0.03	0.90	0.04	1.59	1.03	2.47
	RD	Mild	−0.33	−0.60	−0.06	0.01	0.72	0.55	0.94

No drought used as reference.

**Table 7:** Statistically significant associations between drought and mortality in Ishurdi, 2007–2017.

Drought Index	Mortality Category	Drought Severity	B	Lower	Upper	P	EXP(B)	Lower	Upper
SPI-3	NC	Extreme	0.60	0.05	1.16	0.03	1.83	1.05	3.18
	RD	Extreme	1.11	0.25	1.97	0.01	3.04	1.29	7.15
SPI-12	NC	Mild	−0.22	−0.40	−0.04	0.01	0.80	0.67	0.96
	SU	Mild	−1.05	−1.93	−0.18	0.02	0.35	0.15	0.84
SPEI-3	NC	Extreme	0.85	0.11	1.59	0.02	2.35	1.12	4.93
	RD	Extreme	1.28	0.16	2.39	0.02	3.58	1.18	10.90
SPEI-12	NC	Mild	−0.21	−0.40	−0.03	0.03	0.81	0.67	0.97
	SU	Mild	−1.05	−1.90	−0.19	0.02	0.35	0.15	0.82

No drought used as reference.

**Table 8:** Statistically significant associations between drought and mortality in Rajshahi, 2007–2017.

Drought Index	Mortality Category	Drought Severity	B	Lower	Upper	P	EXP(B)	Lower	Upper
SPI-3	NC	Extreme	−0.51	−0.95	−0.06	0.03	0.60	0.39	0.95
SPI-12	CD	Moderate	−0.49	−0.86	−0.11	0.01	0.61	0.42	0.89
SPEI-3	RD	Mild	−0.24	−0.47	−0.01	0.04	0.79	0.63	0.99
SPEI-12	NC	Moderate	−0.16	−0.31	0.00	0.05	0.85	0.73	1.00
	CD	Moderate	−0.29	−0.55	−0.02	0.04	0.75	0.57	0.98
	SU	Severe	−1.75	−3.22	−0.28	0.02	0.17	0.04	0.75

No drought used as reference.

**Table 9:** Statistically significant associations between drought and mortality in Rangpur, 2007–2017.

Drought Index	Mortality Category	Drought Severity	B	Lower	Upper	P	EXP(B)	Lower	Upper
SPI-3	CD	Mild	−0.25	−0.43	−0.06	0.01	0.78	0.65	0.94
	ID	Moderate	−0.47	−0.93	−0.01	0.05	0.63	0.40	0.99
SPI-12	CD	Moderate	−0.37	−0.70	−0.05	0.03	0.69	0.50	0.96
SPEI-3	NC	Severe	0.24	0.05	0.43	0.01	1.27	1.05	1.53

No drought used as reference.

**Table 10:** Statistically significant associations between drought and mortality in Saidpur, 2007–2017.

Drought Index	Mortality Category	Drought Severity	B	Lower	Upper	P	EXP(B)	Lower	Upper
SPI-3	NC	Extreme	0.64	0.03	1.24	0.04	1.89	1.03	3.47
	ID	Extreme	1.15	0.43	1.86	0.00	3.16	1.54	6.45
SPI-12	NC	Severe	−0.41	−0.78	−0.04	0.03	0.66	0.46	0.96
	RD	Severe	−0.79	−1.48	−0.11	0.02	0.45	0.23	0.90
	SU	Moderate	1.21	0.05	2.36	0.04	3.34	1.05	10.59
SPEI-12	NC	Severe	−0.45	−0.78	−0.11	0.01	0.64	0.46	0.90
	NC	Moderate	−0.58	−0.88	−0.28	0.00	0.56	0.41	0.75
	CD	Moderate	−0.79	−1.29	−0.28	0.00	0.46	0.27	0.75
	RD	Severe	−0.89	−1.54	−0.25	0.01	0.41	0.22	0.78
	RD	Moderate	−0.64	−1.15	−0.13	0.01	0.53	0.32	0.88

No drought used as reference.

## Data Availability

The data presented in this study are available on request from the authors.

## Supplementary Materials

The following are available online at https://www.mdpi.com/article/10.3390/ijerph19063425/s1: Table S1: Bogra significant results with covariates for Model 1, Table S2: Dinajpur significant results with covariates for Model 1, Table S3: Ishurdi significant results with covariates for Model 1, Table S4: Rajshahi significant results with covariates for Model 1, Table S5: Rangpur significant results with covariates for Model 1, Table S6: Saidpur significant results with covariates for Model 1, Table S7: Bogra significant results with covariates for Model 2, Table S8: Ishurdi significant results with covariates for Model 2, Table S9: Rajshahi significant results with covariates for Model 2, Table S10: Rangpur significant results with covariates for Model 2, Table S11: Saidpur significant results with covariates for Model 2.

## References

[1] Rahman H., Alam A. (2016). Forest Dependent Indigenous Communities’ Perception and Adaptation to Climate Change through Local Knowledge in the Protected Area—A Bangladesh Case Study. Climate.

[2] IPCC (2014). Climate Change 2014 Synthesis Report Summary Chapter for Policymakers.

[3] Manyeruke C., Mhandara L. (2013). The Effects of Climate Change and Variability on Food Security in Zimbabwe: A Socio-Economic and Political Analysis. Int. J. Humanit. Soc. Sci..

[4] Wmo Atlas of Mortality and Economic Losses from Weather, Climate and Water Extremes (1970–2019). https://library.wmo.int/doc_num.php?explnum_id=10769.

[5] UN-Water [Internet] Drought Management. http://www.unwater.org/activities/multi-agency-featured-projects/drought-management/en.

[6] Rafiuddin M., Dash B.K., Khanam F. Diagnosis of Drought in Bangladesh using Standardized Precipitation Index. Proceedings of the International Conference on Environment Science and Engineering.

[7] Wilhite D.A., Glantz M.H. (1985). Understanding: The Drought Phenomenon: The Role of Definitions. Water Int..

[8] McKee T.B., Doesken N.J., Kleist J. The relationship of drought frequency and duration to time scales. Proceedings of the 8th Conference on Applied Climatology.

[9] Keka A.I., Matin I., Rahman M., Banu D. (2012). Analysis of Drought in Eastern Part of Bangladesh. Daffodil Int. Univ. J. Sci. Technol..

[10] IPCC (2007). Technical summary of climate change 2007: The physical science basis. Contribution of Working Group I to the Fourth Assessment Report of the Intergovernmental Panel on Climate Change.

[11] He B., Wu J., Lü A., Cui X., Zhou L., Liu M., Zhao L. (2012). Quantitative assessment and spatial characteristic analysis of agricultural drought risk in China. Nat. Hazards.

[12] Smith L., Aragão L.E.O.C., Sabel C., Nakaya T. (2015). Drought impacts on children’s respiratory health in the Brazilian Amazon. Sci. Rep..

[13] Berman J.D., Ebisu K., Peng R.D., Dominici F., Bell M.L. (2017). Drought and the risk of hospital admissions and mortality in older adults in western USA from 2000 to 2013: A retrospective study. Lancet Planet. Health.

[14] Salvador C., Nieto R., Linares C., Díaz J., Gimeno L. (2019). Effects on daily mortality of droughts in Galicia (NW Spain) from 1983 to 2013. Sci. Total Environ..

[15] Salvador C., Nieto R., Linares C., Diaz J., Gimeno L. (2020). Effects of droughts on health: Diagnosis, repercussion, and adaptation in vulnerable regions under climate change. Challenges for future research. Sci. Total Environ..

[16] Salvador C., Nieto R., Linares C., Díaz J., Gimeno L. (2020). Short-term effects of drought on daily mortality in Spain from 2000 to 2009. Environ. Res..

[17] Alam I., Otani S., Majbauddin A., Qing Q., Ishizu S.F., Masumoto T., Amano H., Kurozawa Y. (2021). The Effects of Drought Severity and Its Aftereffects on Mortality in Bangladesh. Yonago Acta Med..

[18] Shahid S. (2010). Recent trends in the climate of Bangladesh. Clim. Res..

[19] Rahman R., Shi Z.H., Chongfa C., Dun Z. (2015). Assessing soil erosion hazard -a raster based GIS approach with spatial principal component analysis (SPCA). Earth Sci. Inform..

[20] Shahid S., Behrawan H. (2008). Drought risk assessment in the western part of Bangladesh, natural hazards. J. Int. Soc. Prev. Mitig. Nat. Hazards.

[21] Paul B.K. (1995). Farmers’ and Public Responses to the 1994–95 Drought in Bangladesh: A Case Study.

[22] Habiba U., Shaw R., Takeuchi Y. (2011). Chapter 2 Socioeconomic Impact of Droughts in Bangladesh. Droughts in Asian Monsoon Region.

[23] Ramamasy S., Baas S. (2007). Climate Variability and Change: Adaptation to Drought in Bangladesh.

[24] Dey N.C., Alam M.S., Sajjan A.K., Bhuiyan M.A., Ghose L., Ibaraki Y., Karim F. (2012). Assessing Environmental and Health Impact of Drought in the Northwest Bangladesh. J. Environ. Sci. Nat. Resour..

[25] OECD (2019). Health Spending (Indicator). https://data.oecd.org/healthres/health-spending.htm.

[26] MoHFW (2017). Bangladesh National Health Accounts 1997–2015.

[27] Owusu P.A., Sarkodie S.A., Pedersen P.A. (2021). Relationship between mortality and health care expenditure: Sustainable assessment of health care system. PLoS ONE.

[28] (2017). Report on Bangladesh Sample Vital Statistics 2017.

[29] Bangladesh. http://www.rangpurdiv.gov.bd/site/page/7be6d41d-18fd-11e7-9461-7.

[30] Bangladesh. http://www.rajshahidiv.gov.bd/site/page/f8d3ea12-1aaf-11e7-8120-.

[31] Bangladesh Meteorological Department. http://www.bmddataportal.com/#/.

[32] Quiring S. (2009). Monitoring Drought: An Evaluation of Meteorological Drought Indices. Geogr. Compass.

[33] Vicente-Serrano S.M., Tomas-Burguera M., Beguería S., Reig-Gracia F., Latorre B., Peña-Gallardo M., Luna M.Y., Morata A., González-Hidalgo J.C. (2017). A High Resolution Dataset of Drought Indices for Spain. Data.

[34] Parsons D.J., Rey D., Tanguy M., Holman I. (2019). Regional variations in the link between drought indices and reported agricultural impacts of drought. Agric. Syst..

[35] Niemeyer S. (2008). New drought indices. Water Manag..

[36] Palmer W.C. (1965). Meteorological Drought.

[37] Vicente-Serrano S.M., Begueria S., Lopez-Moreno J.I. (2010). A multi-scalar drought index sensitive to global warming: The Standardised Precipitation Evapotranspiration Index. J. Clim..

[38] Mishra A.K., Singh V.P. (2010). A review of drought concepts. J. Hydrol..

[39] Zhang Q., Qi T., Singh V.P., Chen Y.D., Xiao M. (2015). Regional Frequency Analysis of Droughts in China: A Multivariate Perspective. Water Resour. Manag..

[40] Tao H., Borth H., Fraedrich K., Su B., Zhu X. (2014). Drought and wetness variability in the Tarim River Basin and connection to large-scale atmospheric circulation. Int. J. Clim..

[41] Zhou Y., Li N., Ji Z., Gu X., Fan B. (2013). Temporal and Spatial Patterns of Droughts Based on Standard Precipitation Index (SPI) in Inner Mongolia during 1981–2010. J. Nat. Resour..

[42] Mathbout S., Lopez-Bustins J.A., Martin-Vide J., Bech J., Rodrigo F.S. (2018). Spatial and temporal analysis of drought variability at several time scales in Syria during 1961–2012. Atmos. Res..

[43] Zhuang S.W., Zuo H.C., Ren P.C., Xiong G.J., Li B.D., Dong W.C., Wang L.Y. (2013). Application of Standardized Precipitation Evapotranspiration Index in China. Clim. Environ. Res..

[44] Beguería S., Vicente-Serrano S.M. (2013). Package ‘SPEI’ v 1.4: Calculation of the Standardised Precipitation-Evapotranspiration Index. https://cran.r-project.org/web/packages/SPEI/SPEI.Pdf.

[45] Páscoa P., Gouveia C.M., Russo A., Trigo R. (2017). Drought Trends in the Iberian Peninsula over the Last 112 Years. Adv. Meteorol..

[46] Ortiz-Gómez R., De Zacatecas Z.U.A., Cardona-Díaz J.C., Ortiz-Robles F.A., Alvarado-Medellin P. (2018). Caracterización de las sequías mediante la comparación de tres índices multiescalares en Zacatecas, México. Tecnol. Cienc. Agua.

[47] Agnew C.T. (2000). Using the SPI to Identify Drought [Internet]. Drought Network News (1994–2001). http://digitalcommons.unl.edu/droughtnetnews/1.

[48] Salvador C., Nieto R., Linares C., Díaz J., Alves C., Gimeno L. (2021). Drought effects on specific-cause mortality in Lisbon from 1983 to 2016: Risks assessment by gender and age groups. Sci. Total Environ..

[49] Stanke C., Kerac M., Prudhomme C., Medlock J., Murray V. (2013). Health Effects of Drought: A Systematic Review of the Evidence. PLoS Curr..

[50] Bandyopadhyay N., Bhuiyan C., Saha A.K. (2016). Heat waves, temperature extremes and their impacts on monsoon rainfall and meteorological drought in Gujarat, India. Nat. Hazards.

[51] Spinoni J., Vogt J.V., Naumann G., Barbosa P., Dosio A. (2018). Will drought events become more frequent and severe in Europe?. Int. J. Climatol..

[52] Yusa A., Berry P., Cheng J.J., Ogden N., Bonsal B., Stewart R., Waldick R. (2015). Climate Change, Drought and Human Health in Canada. Int. J. Environ. Res. Public Health.

[53] Hashizume M., Wagatsuma Y., Hayashi T., Saha S.K., Streatfield K., Yunus M. (2009). The effect of temperature on mortality in rural Bangladesh—A population-based time-series study. Int. J. Epidemiol..

[54] Burkart K., Khan M.H., Krämer A., Breitner S., Schneider A., Endlicher W.R. (2011). Seasonal variations of all-cause and cause-specific mortality by age, gender, and socioeconomic condition in urban and rural areas of Bangladesh. Int. J. Equity Health.

[55] Alam N., Lindeboom W., Begum D., Streatfield P.K. (2012). The association of weather and mortality in Bangladesh from 1983–2009. Glob. Health Action.

[56] Burkart K., Schneider A., Breitner S., Khan M.H., Krämer A., Endlicher W. (2011). The effect of atmospheric thermal conditions and urban thermal pollution on all-cause and cardiovascular mortality in Bangladesh. Environ. Pollut..

[57] (2007). Housing, Energy and Thermal Comfort: A Review of 10 Countries within the WHO European Region. https://www.euro.who.int/__data/assets/pdf_file/0008/97091/E89887.pdf.

[58] Preliminary Report on Economic Census 2013. http://bbs.portal.gov.bd/sites/default/files/files/bbs.portal.gov.bd/page/f2661853_b857_49c5_8761_e1641e3aec9b/Pre_Report_Econo_Cen_13.pdf.

[59] Wilhite D.A., Wilhite D. (2000). Drought as a natural hazard: Concepts and definitions. Drought: A Global Assessment.

[60] Wilhite D.A., Svoboda M.D., Hayes M.J. (2007). Understanding the complex impacts of drought: A key to enhancing drought mitigation and preparedness. Water Resour. Manag..

[61] Ebi K.L., Bowen K. (2016). Extreme events as sources of health vulnerability: Drought as an example. Weather. Clim. Extrem..

