# Supplementary material for: Short- and Long-Term Effects of Drought on Selected Causes of Mortality in Northern Bangladesh
Source: Int J Environ Res Public Health. 2022 Mar 14;19(6):3425. doi: 10.3390/ijerph19063425 (PMC8951054; doi:10.3390/ijerph19063425)
Supplement: Supplementary file 1 [file ijerph-19-03425-s001.zip › ijerph-1593839-supplementary.pdf]

Table S1. Bogra significant results with covariates for Model 1

Drought Index	Mortality Category	Factors	B	Lower	Upper	P	EXP(B)	Lower	Upper
**3SPI**	NC	temp	-0.03	-0.05	-0.01	0.00	0.97	0.95	0.99
	CD	temp	-0.04	-0.08	-0.01	0.01	0.96	0.92	0.99
	ID	temp	-0.06	-0.11	-0.01	0.01	0.94	0.90	0.99
	ID	humidity	0.04	0.01	0.07	0.01	1.04	1.01	1.07
**12SPI**	NC	temp	-0.04	-0.05	-0.02	0.00	0.96	0.95	0.98
	CD	temp	-0.04	-0.07	0.00	0.03	0.96	0.93	1.00
	RD	temp	-0.04	-0.07	0.00	0.03	0.96	0.93	1.00
	ID	temp	-0.07	-0.12	-0.02	0.00	0.93	0.89	0.98
	ID	humidity	0.03	0.00	0.06	0.03	1.03	1.00	1.07
**3SPEI**	NC	temp	-0.04	-0.05	-0.02	0.00	0.96	0.95	0.98
	CD	temp	-0.04	-0.07	-0.01	0.02	0.96	0.93	0.99
	RD	temp	-0.04	-0.07	0.00	0.02	0.96	0.93	1.00
	ID	temp	-0.07	-0.12	-0.02	0.01	0.93	0.89	0.98
	ID	humidity	0.04	0.00	0.07	0.03	1.04	1.00	1.07
**12SPEI**	NC	temp	-0.04	-0.05	-0.02	0.00	0.96	0.95	0.98
	CD	temp	-0.04	-0.07	-0.01	0.01	0.96	0.93	0.99
	RD	temp	-0.03	-0.07	0.00	0.05	0.97	0.93	1.00
	ID	temp	-0.07	0.03	-0.12	0.01	0.94	0.89	0.99

[1]No drought used as reference. *NC, natural cause, CD, cardiovascular disease, RD, respiratory disease, ID, infectious disease, SU, suicide, temp, temperature.

Table S2: Dinajpur significant results with covariates for Model 1

Drought Index	Mortality Category	Factors	B	Lower	Upper	P	EXP(B)	Lower	Upper
**3SPI**	NC	temp	-0.03	-0.05	-0.01	0.00	0.97	0.95	0.99
	CD	humidity	-0.02	-0.04	0.00	0.03	0.96	0.93	1.00
	RD	temp	-0.04	-0.07	0.00	0.03	1.04	1.00	1.07
	ID	humidity	0.04	0.00	0.07	0.03	1.04	1.00	1.07
**12SPI**	NC	temp	-0.03	-0.05	-0.01	0.00	0.97	0.95	0.99
	CD	humidity	-0.02	-0.04	0.00	0.03	0.98	0.96	1.00
	ID	humidity	0.04	0.00	0.07	0.03	1.04	1.00	1.07
**3SPEI**	NC	temp	-0.03	-0.05	-0.01	0.00	0.97	0.95	0.99
	RD	temp	-0.03	-0.06	0.00	0.03	0.97	0.94	1.00
	ID	humidity	0.03	0.00	0.06	0.03	1.03	1.00	1.07
**12SPEI**	NC	temp	-0.03	-0.05	-0.01	0.01	0.97	0.95	0.99
	CD	humidity	-0.02	-0.04	0.00	0.03	0.98	0.96	1.00
	ID	humidity	0.04	0.00	0.07	0.03	1.04	1.00	1.07

[1]No drought used as reference. *NC, natural cause, CD, cardiovascular disease, RD, respiratory disease, ID, infectious disease, SU, suicide, temp, temperature

Table S3: Ishurdi significant results with covariates for Model 1

Drought Index	Mortality Category	Factors	B	Lower	Upper	P	EXP(B)	Lower	Upper
**3SPI**	NC	temp	-0.04	-0.06	-0.02	0.00	0.96	0.94	0.98
	CD	temp	-0.04	-0.07	-0.02	0.00	0.96	0.93	0.98
	RD	temp	-0.05	-0.09	-0.01	0.01	0.95	0.92	0.99
**12SPI**	NC	temp	-0.04	-0.06	-0.02	0.00	0.96	0.95	0.98
	CD	temp	-0.04	-0.07	-0.01	0.01	0.96	0.94	0.99
	RD	temp	-0.05	-0.09	-0.01	0.02	0.95	0.92	0.99
**3SPEI**	NC	temp	-0.03	-0.05	-0.01	0.00	0.97	0.95	0.99
	CD	temp	-0.04	-0.07	-0.01	0.01	0.96	0.93	0.99
	RD	temp	-0.04	-0.08	0.00	0.04	0.96	0.92	1.00
**12SPEI**	NC	temp	-0.04	-0.06	-0.02	0.00	0.97	0.95	0.98
	CD	temp	-0.04	-0.07	-0.01	0.01	0.96	0.94	0.99
	RD	temp	-0.05	-0.09	-0.01	0.02	0.95	0.92	0.99

[1]No drought used as reference. *NC, natural cause, CD, cardiovascular disease, RD, respiratory disease, ID, infectious disease, SU, suicide, temp, temperature

Table S4: Rajshahi significant results with covariates for Model 1

Drought Index	Mortality Category	Factors	B	Lower	Upper	P	EXP(B)	Lower	Upper
**3SPI**	NC	temp	-0.04	-0.05	-0.03	0.00	0.96	0.95	0.97
	CD	temp	-0.04	-0.06	-0.01	0.00	0.96	0.94	0.99
	RD	temp	-0.07	-0.09	-0.05	0.00	0.94	0.91	0.96
	ID	temp	-0.05	-0.08	-0.02	0.00	0.95	0.92	0.98
	SU	temp	0.10	0.01	0.19	0.03	1.10	1.01	1.21
**12SPI**	NC	temp	-0.04	-0.05	-0.03	0.00	0.96	0.95	0.98
	CD	temp	-0.03	-0.05	-0.01	0.00	0.97	0.95	0.99
	RD	temp	-0.06	-0.08	-0.04	0.00	0.94	0.92	0.96
	ID	temp	-0.05	-0.08	-0.03	0.00	0.95	0.92	0.97
	SU	temp	0.11	0.02	0.20	0.02	1.11	1.02	1.22
**3SPEI**	NC	temp	-0.04	-0.05	-0.03	0.00	0.96	0.95	0.97
	CD	temp	-0.03	-0.05	-0.01	0.00	0.97	0.95	0.99
	RD	temp	-0.06	-0.08	-0.04	0.00	0.94	0.92	0.96
	ID	temp	-0.05	-0.08	-0.03	0.00	0.95	0.92	0.97
	SU	temp	0.10	0.02	0.19	0.02	1.11	1.02	1.21
**12SPEI**	NC	temp	-0.04	-0.05	-0.03	0.00	0.96	0.95	0.97
	CD	temp	-0.04	-0.06	-0.02	0.00	0.96	0.94	0.98
	RD	temp	-0.06	-0.09	-0.04	0.00	0.94	0.92	0.96
	ID	temp	-0.06	-0.09	-0.03	0.00	0.94	0.92	0.97
	ID	humidity	-0.02	-0.03	0.00	0.03	0.98	0.97	1.00
	SU	temp	0.10	0.01	0.19	0.03	1.11	1.01	1.21

[1]No drought used as reference. *NC, natural cause, CD, cardiovascular disease, RD, respiratory disease, ID, infectious disease, SU, suicide, temp, temperature

Table S5: Rangpur significant results with covariates for Model 1

Drought Index	Mortality Category	Factors	B	Lower	Upper	P	EXP(B)	Lower	Upper
**3SPI**	NC	temp	-0.04	-0.06	-0.03	0.00	0.96	0.95	0.97
	CD	temp	-0.05	-0.07	-0.03	0.00	0.95	0.93	0.97
	RD	temp	-0.05	-0.08	-0.02	0.00	0.95	0.92	0.98
**12SPI**	NC	temp	-0.04	-0.06	-0.03	0.00	0.96	0.95	0.98
	CD	temp	-0.04	-0.07	-0.02	0.00	0.96	0.93	0.98
**3SPEI**	NC	temp	-0.04	-0.05	-0.02	0.00	0.96	0.95	0.98
	CD	temp	-0.04	-0.07	-0.02	0.00	0.96	0.93	0.98
	RD	temp	-0.05	-0.08	-0.02	0.00	0.95	0.93	0.98
**12SPEI**	NC	temp	-0.04	-0.06	-0.02	0.00	0.96	0.95	0.98
	CD	temp	-0.04	-0.07	-0.02	0.00	0.96	0.93	0.98
	RD	temp	-0.05	-0.07	-0.02	0.00	0.95	0.93	0.98

[1]No drought used as reference. *NC, natural cause, CD, cardiovascular disease, RD, respiratory disease, ID, infectious disease, SU, suicide, temp, temperature

Table S6. Syedpur significant results with covariates for Model 1

Drought Index	Mortality Category	Factors	B	Lower	Upper	P	EXP(B)	Lower	Upper
**3SPI**	NC	temp	-0.05	-0.07	-0.02	0.00	0.96	0.93	0.98
	CD	temp	-0.04	-0.07	0.00	0.04	0.97	0.93	1.00
	RD	temp	-0.06	-0.09	-0.02	0.00	0.94	0.91	0.98
	ID	temp	-0.05	-0.10	-0.01	0.02	0.95	0.91	0.99
	SU	Humidity	0.09	0.00	0.18	0.04	1.10	1.00	1.20
**12SPI**	NC	temp	-0.05	-0.07	-0.02	0.00	0.95	0.93	0.98
	RD	temp	-0.06	-0.09	-0.02	0.00	0.94	0.91	0.98
	ID	temp	-0.06	-0.11	-0.01	0.03	0.94	0.90	0.99
**3SPEI**	NC	temp	-0.05	-0.07	-0.03	0.00	0.95	0.93	0.97
	CD	temp	-0.04	-0.07	0.00	0.04	0.96	0.93	1.00
	RD	temp	-0.07	-0.10	-0.03	0.00	0.94	0.90	0.97
	ID	temp	-0.07	-0.12	-0.02	0.01	0.94	0.89	0.98
	NC	temp	-0.04	-0.07	-0.02	0.00	0.96	0.94	0.98
	RD	temp	-0.06	-0.09	-0.02	0.00	0.94	0.91	0.98

[1]No drought used as reference. *NC, natural cause, CD, cardiovascular disease, RD, respiratory disease, ID, infectious disease, SU, suicide, temp, temperature

Table S7: Bogra significant results with covariates for Model 2

Drought Index	Mortality Category	Factors	B	Lower	Upper	P	EXP(B)	Lower	Upper
**3SPI**	NC	Humidity	0.01	0.00	0.03	0.06	1.01	1.00	1.03
	NC	THE ($)	-0.09	-0.14	-0.05	0.00	0.91	0.87	0.96
	RD	THE ($)	-0.14	-0.24	-0.04	0.01	0.87	0.78	0.97
**12SPI**	NC	THE ($)	-0.10	-0.15	-0.05	0.00	0.90	0.86	0.95
	RD	THE ($)	-0.16	-0.26	-0.06	0.00	0.85	0.77	0.94
**3SPEI**	NC	Humidity	0.01	0.00	0.03	0.05	1.01	1.00	1.03
	NC	THE ($)	-0.09	-0.14	-0.05	0.00	0.91	0.87	0.95
	RD	THE ($)	-0.16	-0.27	-0.06	0.00	0.85	0.77	0.94
**12SPEI**	NC	THE ($)	-0.10	-0.15	-0.05	0.00	0.90	0.86	0.95
	RD	THE ($)	-0.16	-0.26	-0.05	0.00	0.86	0.77	0.95

[1]No drought used as reference. *NC, natural cause, CD, cardiovascular disease, RD, respiratory disease, ID, infectious disease, SU, suicide, temp, temperature, THE, total health expenditure.

Table S8: Ishurdi significant results with covariates for Model 2

Drought Index	Mortality Category	Factors	B	Lower	Upper	P	EXP(B)	Lower	Upper
**3SPI**	NC	THE ($)	-0.08	-0.14	-0.02	0.01	0.92	0.87	0.98
	RD	THE ($)	-0.13	-0.26	-0.01	0.03	0.87	0.77	0.99
	ID	THE ($)	-0.19	-0.36	-0.01	0.04	0.83	0.70	0.99
**12SPI**	NC	THE ($)	-0.10	-0.16	-0.04	0.00	0.90	0.85	0.96
	CD	temp	-0.07	-0.14	0.00	0.05	0.93	0.87	1.00
	CD	THE ($)	-0.11	-0.20	-0.02	0.02	0.90	0.82	0.98
	ID	temp	-0.14	-0.26	-0.02	0.02	0.87	0.77	0.98
	ID	THE ($)	-0.19	-0.36	-0.03	0.02	0.82	0.70	0.97
**3SPEI**	NC	THE ($)	-0.08	-0.14	-0.01	0.02	0.93	0.87	0.99
	RD	THE ($)	-0.14	-0.27	-0.01	0.03	0.87	0.77	0.99
	ID	temp	-0.13	-0.25	-0.01	0.03	0.88	0.78	0.99
	ID	THE ($)	-0.18	-0.33	-0.03	0.02	0.83	0.72	0.97
**12SPEI**	NC	THE ($)	-0.09	-0.15	-0.02	0.01	0.92	0.86	0.98
	CD	THE ($)	-0.10	-0.19	0.00	0.04	0.91	0.83	1.00
	ID	temp	-0.14	-0.26	-0.03	0.02	0.87	0.77	0.98
	ID	THE ($)	-0.18	-0.34	-0.01	0.04	0.84	0.71	0.99

[1]No drought used as reference. *NC, natural cause, CD, cardiovascular disease, RD, respiratory disease, ID, infectious disease, SU, suicide, temp, temperature, THE, total health expenditure

Table S9. Rajshahi significant results with covariates for Model 2

Drought Index	Mortality Category	Factors	B	Lower	Upper	P	EXP(B)	Lower	Upper
**3SPI**	NC	Temperature	-0.05	-0.08	-0.02	0.00	0.95	0.92	0.98
	NC	THE ($)	-0.04	-0.08	0.00	0.05	0.96	0.92	1.00
	CD	THE ($)	-0.07	-0.14	0.00	0.04	0.93	0.87	1.00
	RD	Temp	-0.16	-0.21	-0.10	0.00	0.85	0.81	0.90
	ID	Temp	-0.09	-0.16	-0.02	0.01	0.91	0.85	0.98
	ID	Humidity	-0.04	-0.07	-0.01	0.01	0.96	0.94	0.99
**12SPI**	NC	Temp	-0.06	-0.09	-0.03	0.00	0.94	0.92	0.97
	CD	THE ($)	-0.07	-0.14	-0.01	0.03	0.93	0.87	0.99
	RD	Temp	-0.16	-0.21	-0.10	0.00	0.86	0.81	0.91
	ID	Temp	-0.10	-0.17	-0.03	0.00	0.91	0.85	0.97
	ID	Humidity	-0.04	-0.07	-0.01	0.00	0.96	0.94	0.99
**3SPEI**	NC	Temperature	-0.05	-0.08	-0.02	0.00	0.95	0.92	0.98
	NC	Humidity	-0.01	-0.02	0.00	0.03	0.99	0.98	1.00
	NC	THE ($)	-0.04	-0.08	0.00	0.05	0.96	0.92	1.00
	CD	THE ($)	-0.07	-0.14	-0.01	0.03	0.93	0.87	0.99
	RD	Tempe	-0.15	-0.21	-0.09	0.00	0.86	0.81	0.91
	ID	Temperature	-0.10	-0.16	-0.03	0.01	0.91	0.85	0.97
	ID	Humidity	-0.04	-0.07	-0.01	0.01	0.96	0.93	0.99
**12SPEI**	NC	Temperature	-0.06	-0.09	-0.03	0.00	0.94	0.92	0.97
	NC	Humidity	-0.01	-0.02	0.00	0.04	0.99	0.98	1.00
	CD	THE ($)	-0.07	-0.13	0.00	0.05	0.94	0.88	1.00
	RD	Temp	-0.16	-0.22	-0.10	0.00	0.85	0.81	0.90
	ID	Temperature	-0.11	-0.18	-0.05	0.00	0.89	0.83	0.95
	ID	Humidity	-0.04	-0.07	-0.02	0.00	0.96	0.93	0.99
	ID	THE ($)	-0.10	-0.21	0.00	0.05	0.90	0.81	1.00

[1]No drought used as reference. *NC, natural cause, CD, cardiovascular disease, RD, respiratory disease, ID, infectious disease, SU, suicide, temp, temperature, THE, total health expenditure

Table S10: Rangpur significant results with covariates for Model 2

Drought Index	Mortality Category	Factors	B	Lower	Upper	P	EXP(B)	Lower	Upper
**3SPI**	NC	Temp	-0.05	-0.08	-0.01	0.01	0.95	0.92	0.99
	CD	Temp	-0.06	-0.11	-0.01	0.02	0.94	0.89	0.99
**12SPI**	NC	Temp	-0.05	-0.08	-0.01	0.01	0.96	0.92	0.99
	RD	THE ($)	0.15	0.01	0.30	0.04	1.17	1.01	1.35
**3SPEI**	NC	Temp	-0.05	-0.08	-0.01	0.01	0.96	0.92	0.99
	RD	THE ($)	0.15	0.01	0.28	0.04	1.16	1.01	1.33
	SU	THE ($)	-0.44	-0.86	-0.03	0.04	0.64	0.42	0.97
**12SPEI**	NC	Temp	-0.05	-0.08	-0.01	0.01	0.96	0.92	0.99
	RD	THE ($)	0.16	0.01	0.31	0.03	1.17	1.01	1.36

[1]No drought used as reference. *NC, natural cause, CD, cardiovascular disease, RD, respiratory disease, ID, infectious disease, SU, suicide, temp, temperature, THE, total health expenditure

Table S11: Syedpur significant results with covariates for Model 2

Drought Index	Mortality Category	Factors	B	Lower	Upper	P	EXP(B)	Lower	Upper
**3SPI**	NC	Temp	-0.05	-0.10	0.00	0.04	0.95	0.90	1.00
**12SPI**	NC	Temp	-0.06	-0.11	-0.01	0.03	0.94	0.90	0.99
**3SPEI**	NC	Temp	-0.06	-0.11	-0.01	0.02	0.94	0.89	0.99
**12SPEI**	NC	Temp	-0.05	-0.10	0.00	0.03	0.95	0.90	1.00

[1]No drought used as reference. *NC, natural cause, CD, cardiovascular disease, RD, respiratory disease, ID, infectious disease, SU, suicide, temp, temperature, THE, total health expenditure
